# The exopolyphosphatase TbrPPX1 of *Trypanosoma brucei*

**DOI:** 10.1186/1471-2180-11-4

**Published:** 2011-01-06

**Authors:** Edith Luginbuehl, Stefan Kunz, Laurent Wentzinger, Florian Freimoser, Thomas Seebeck

**Affiliations:** 1 Institute of Cell Biology, University of Bern, Baltzerstrasse 4, CH-3012 Bern, Switzerland; 2 Institute of Plant Sciences, ETH Zurich, Universitaetsstrasse 2, CH-8092 Zurich

## Abstract

**Background:**

Exopolyphosphatases and pyrophosphatases play important but still incompletely understood roles in energy metabolism, and also in other aspects of cell biology such as osmoregulation or signal transduction. Earlier work has suggested that a human exopolyphosphatase, Prune, might exhibit cyclic nucleotide phosphodiesterase activity.

**Results:**

The kinetoplastida, a large order of unicellular eukaryotes that contains many important pathogens such as *Trypanosoma brucei *(human sleeping sickness), *Trypanosoma cruzi *(Chagas disease) or *Leishmania *ssp (several clinically dinstinct leishmaniases) all contain several exo- and pyrophosphatases. The current study provides a systematic classification of these enzymes, which now allows to situate the information that is already available on some of these enzymes. It then analyses the exopolyphosphatase TbrPPX1 of *T. brucei *in detail, using RNA interference and genetic knockouts in an attempt to define its function, and immunofluorescence microscopy to study its subcellular localization.

TbrPPX1 is an exopolyphosphatase that does hydrolyze pentasodium triphosphate, but not organic triphosphates such as ATP, pyrophosphate or long-chain polyphosphates. Finally, the study investigates the potential cyclic nucleotide phosphodiesterase activity of TbrPPX1.

**Conclusions:**

All kinetoplastid genomes that are currently available contain genes for an exopolyphosphatase and two classes of pyrophosphatases, one associated with the acidocalcisomes and one cytoplasmic. TbrPPX1 represents the *T. brucei *exopolyphosphatase. It is located throughout the cytoplasm, and its genetic ablation does not produce a dramatic phenotype. Importantly, TbrPPX1 does not exhibit any cyclic nucleotide specific phosphodiesterase activity, which definitively eliminates it as an additional player in cAMP signalling of the kinetoplastida.

## Background

Inorganic polyphosphates and the exopolyphosphatases/pyrophosphatases involved in their hydrolysis play an important role in the phosphate and energy metabolism of all living organisms [1,2]. The polyphosphates, linear polymers ranging from two to hundreds of phosphate residues linked by high-energy phosphoanhydride bonds, are mostly concentrated in specialized organelles, the volutin granules or acidocalcisomes [1,3,4]. They serve as osmotically inert phosphate and energy stores that also contain high concentrations of divalent cations and basic amino acids. Hydrolysis by polyphosphatases and pyrophosphatases provides phosphate in periods of phosphate limitation [1] or to control osmotic stress [3,5]. Besides these roles that require massive amounts of polyphosphates, both molecular species, polyphosphates and pyrophosphate, may also exert more subtle cytosolic functions, such as e.g. gating the cystic fibrosis transmembrane conductance regulator [6].

The polyphosphatases belong to the large superfamily of the DHH phosphoesterases [7]. This superfamily is divided into two subfamilies that share four N terminal signature motifs. They differ in their C-terminal moieties where subfamily 2 carries two additional conserved motifs. Subfamily 1 includes the bacterial RecJ nucleases, while subfamily 2 members fall into three functional groups, the pyrophosphatases, the exopolyphosphatases and the closely related "prune-type" exopolyphosphatases. The exopolyphosphatase/pyrophosphatase groups and the prune group can be readily distinguished since members of the former group carry the sequences DHN and DHH in their motifs II and III, respectively, while all prunes carry the sequences DHH and DHR at the respective positions [8]. Within the prune group, vertebrate prunes are distinguished from their non-vertebrate homologues by the acquisition of a C-terminal extension of about 80 amino acids [9]. This region contains a proline-rich and a helical domain which are essential for the physical interaction of human prune with nucleoside diphosphate kinase A (nm23-H1) and glycogen synthase kinase 3b [10]. Human prune is a short-chain selective exopolyphosphatase that preferentially hydrolyzes tri- and tetrapolyphosphates, as well as nucleoside 5'-tetraphosphates [9].

The kinetoplastids, a group of unicellular eukaryotes that comprises many important pathogens, contain prominent polyphosphate storage organelles, the acidocalcisomes. These acidic, electron-dense compartments contain the major part of the cellular polyphosphate, as well as high concentrations of calcium, magnesium, sodium, potassium, zinc, iron and the basic amino acids arginine and lysine [3,4]. The polyphosphate content of the acidocalcisomes changes rapidly under conditions of hyper- or hypoosmotic stress [11]. In *T. brucei*, an acidocalcisomal pyrophosphatase TbVSP1 was characterized [12] and a series of inhibitors against this enzyme were developed [13]. This pyrophosphatase preferentially hydrolyzes inorganic pyrophosphate, with gradually decreasing activity against polyphosphates of higher chain lengths. In *L. major*, an exopolyphosphatase, LmPPX, was identified which exhibited a preference for short-chain polyphosphates. This enzyme appears to be located both in the cytosol and in the acidocalcisomes [14]. Similar results were also obtained with its homologue of *T. cruzi*, TcPPX [15]. This enzyme does not hydrolyze long-chain inorganic polyphosphates or ATP. It is highly active against polyphosphates of short chain length (tri- or tetraphosphates), with strongly decreasing activity for longer chain polyphosphates. Overexpression of the enzyme delayed the regulatory volume decrease after hypoosmotic shock, suggesting that it may play a role in osmoregulation. The selectivity of all known kinetoplastid polyphosphatases for short chain polyphosphates is in line with the observation that the average polyphosphate chain length in these organisms is only 3 to 4 residues [3]. A preliminary report also documented the recombinant expression and refolding of a *T. brucei *exopolyphosphatase and provided initial data on its activity [16].

The current study provides a general overview over the pyrophosphatases and exopolyphosphatases of the kinetoplastida, and it identifies, localizes and characterizes the exopolyphosphatase TbrPPX1 from *T. brucei*. Furthermore, it demonstrates that TbrPPX1 does not contain a cyclic-nucleotide specific phosphodiesterase activity, as had been reported earlier for the human prune enzyme [17].

## Results

### Identification of exopolyphosphatases and pyrophosphatases in the kinetoplastids

**Figure 1:**
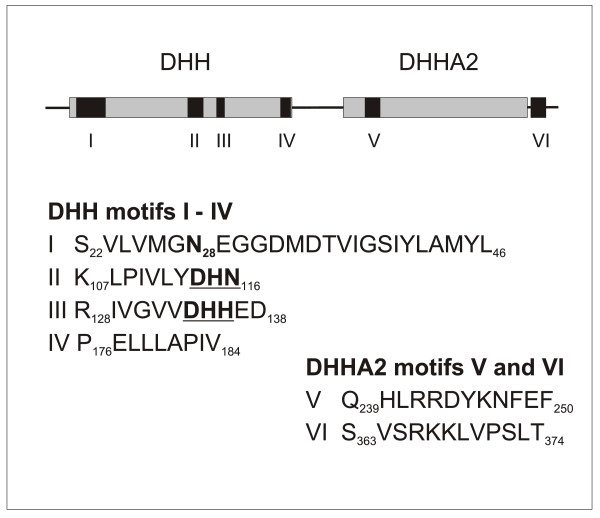
**TbrPPX1 is a predicted exopolyphosphatase that belongs to the subfamily 2 of the DHH superfamily**. Dark boxes: motifs I - IV and V - VI of the DHH and the DHHA2 domains, respectively. Amino acid numbering corresponds to the TbrPPX1 sequence. Bold, underlined: active site motifs that discriminate the prune subfamily (DHH and DHR in motifs II and III, respectively) from the exopolyphosphatases/pyrophosphatases (DHN and DHH in motifs II and III, respectively). For a discussion of the functional consequences of his shift of the DHH signature from motif II to motif III see [8].

TbrPPX1 was identified by blastp searching of the *T. brucei *database with the amino acid sequence of human prune [GenBank:NP_067045]. A single copy gene [GeneDB:Tb09.160.1950; UniProt/TrEMBL: Q7Z032] was identified on chromosome 9 (e value 5 × 10^-17^). TbrPPX1 is a polypeptide of 383 amino acids with a predicted molecular mass of 42866 Da and a pI of 5.39. The polypeptide contains a DHH domain (amino acids 16-184) and a DHHA2 domain (amino acids 222-377) that identify it as a member of the DHH superfamily. The DHH domain contains the characteristic four motifs I - IV, while domain DHHA2 contains the two additional motifs V and VI that identify TbrPPX1 as a member of subfamily 2 of the DHH superfamily (Figure 1). TbrPPX1 is predicted to be a exopolyphosphatase due to the presence of the conserved motif G_27_NEGG_31_[8]. All exopolyphosphatases carry an asparagine in the position corresponding to N_28 _of TbrPPX1, while this residue is replaced by a histidine in the pyrophosphatases. This histidine is part of the first of two metal binding domains in pyrophosphatases. Furthermore, in motifs II and III, TbrPPX1 contains the sequence motifs DHN and DHH, respectively, which set it apart from the prune subfamily that contains the motifs DHH and DHR at the respective positions [8]. Characteristically, TbrPPX1 also lacks the C-terminal extension of about 80 amino acids that is present in all vertebrate prunes, but is absent from the invertebrate prune homologues [9] and from the exopolyphosphatases.

**Figure 2:**
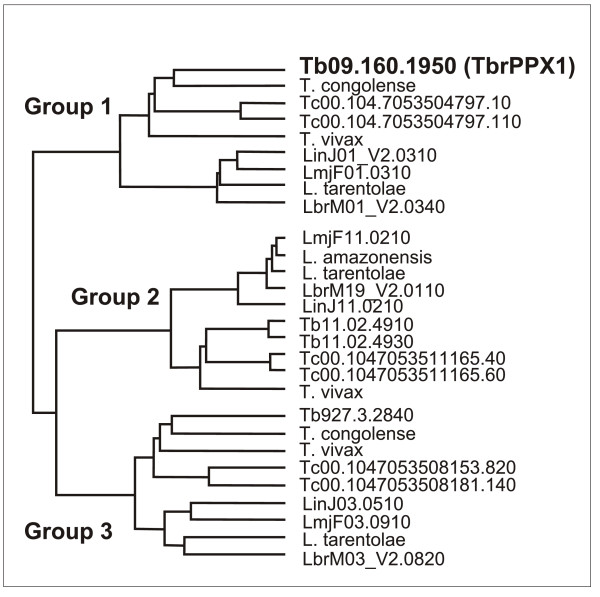
**Neighbour distance tree of amino acid sequences of the kinetoplastid exo-and endopolyphosphatases**. Group 1: cytosolic exopolyphosphatases; group: acidocalcisomal inorganic pyrophosphatases; group 3: pyrophosphatases. For the designations of the individual genes and proteins see Table 1.

Blast searching of the genomic databases of *T. congolense*, *T. vivax*, *T. cruzi*, *L. major*, *L. infantum, L. brasiliensis *and *L. tarentolae *with TbrPPX1 demonstrated the presence of one orthologue of TbrPPX1 (three for *T. cruzi*) in each genome (Figure 2 and Table 1). The identical set of genes was also retrieved when searching the databases with the *S. cerevisiae *exopolyphosphatase ScPPX1 [GenBank: AAB68368]. All these TbrPPX1 homologues (group 1) share extensive sequence conservation and consist of about 380 amino acids, with calculated isoelectric points of about 5.5. For several of them, an exopolyphosphatase activity has been experimentally demonstrated [[14,15], this study].

**Table 1:** The exopolyphosphatases/pyrophosphatases of the kinetoplastids

Organism	GeneDB	TrEMBL	Gene ID	Amino acids	Calc. MW	Calc. pI	**Ref**.
Group 1 (exopoly-phosphatases)							

T. brucei	Tb09.160.1950 (TbrPPX1)	Q7Z032	3660027	383	42865	5.39	[16], this study
T. congolense	congo940f01.q1k_0	---		383	43004	5.66	
T. cruzi	Tc00.1047053504797.10	Q4DJ30	3545900	383	43029	5.95	15
	Tc00.1047053511577.110	Q6Y656		383	43121	5.96	
T. vivax	tviv676c08.p1k_16	---		382	43434	5.68	
L. braziliensis	LbrM01_V2.0340	A4H355	5412361	387	42862	5.80	
L. infantum	LinJ01_V3.0310	A4HRF2	5066108	387	42626	5.59	
L. major	LmjF01.0310	Q25348	800604	388	42595	5.63	[14]
L. tarentolae	r1596.contig3320-2-1007-2215	---		387	43035	5.74	

Group 2 (acidocalcisomal pyrophosphatases)							

T. brucei	Tb11.02.4910	Q384W5	3665799	414	47330	5.73	[12,13]
	Tb11.02.4930	Q7Z029		414	47307	5.70	
T. cruzi	Tc00.1047053511165.40	Q4D5E4	3540239	414	47793	5.71	
	Tc00.1047053503613.60	Q4JH30	3537396	414	47854	5.71	
T. vivax	Tviv426a04.q1k_3	---		414	47727	5.75	
L. braziliensis	LbrM19_V2.0110	A4H9T7	5414648	443	51256	5.51	
L. infantum	LinJ11.0210	A4HUT3	5067199	412	47390	5.52	
L. major	LmjF11.0210	Q4QH59	5649763	443	50994	5.38	
L. tarentolae	r1596.contig1511-1-4543-5877	---		443	51075	5.40	
L. amazonensis	---	Q7Z031		443	51175	5.32	[35]

Group 3 (cytosolic pyrophosphatases)		---					

T. brucei	Tb927.3.2840	Q57ZM8	3656220	261	28676	5.66	
T. congolense	congo1253h06.p1k_11			262	29016	5.67	
T. cruzi	Tc00.1047053508153.820	Q4E611	3555184	276	31146	5.76	
	Tc00.1047053508181.140	Q4DR95	3548870	271	30554	6.12	
T. vivax	tviv222a06.p1k_8	---		263	26220	5.15	
L. braziliensis	LbrM03_V2.0820	A4H3Q3	5412574	269	29744	5.90	
L. infantum	LinJ03.0510	A4HRX7	5066310	226	25108	5.15	
L. major	LmjF03.0910	Q9N640	809741	226	24973	5.41	
L. tarentolae	r1596.contig6751-4-7549-6743	---		263	28971	5.83	

Group 1 contains the exopolyphosphatases, and group 2 consists of the acidocalcisomal inorganic pyrophosphatases. For both groups, the activities of representative members have been experimentally determined. Group 3 represents a homogeneous group of predicted, putatively cytosolic inorganic pyrophosphatases for which no experimental data are available so far. Designations are by gene name (TriTrypDB), by the TrEMBL database nomenclature and by gene identification number (where available). Total amino acid numbers and calculated molecular mass and pI values are also given.

Analysis of the kinetoplastid genomes for the presence of additional poly- or pyrophosphatases resulted in the identification of two additional groups (Figure 2). Group 2 represents the kinetoplastid-specific acidocalcisomal pyrophosphatases, one of which [GeneDB: Tb11.02.4930] has been experimentally characterized [12,13]. Their lengths vary from 414 to 443 amino acids, with isoelectric points between 5.3 and 5.8. They are all characterized by an inorganic pyrophosphatase domain [InterPro: IPR008162] which, in Tb11.02.4930 extends from amino acids 225 to 404. Finally, group 3 represents yet uncharacterized, putatively cytosolic pyrophosphatases, with lengths from 260 to 320 amino acids and pIs varying from 5.2 to 6.3. Their sequences also contain the inorganic pyrophosphatase domain, extending from about amino acids 67 to 247. Interestingly, no recognizable genes coding for endopolyphosphatases were detected in any of the kinetoplastid genomes.

### Expression and subcellular localization of TbrPPX1

RT-PCR and Northern blotting demonstrated that the *TbrPPX1 *gene is expressed at similar levels both in bloodstream and in procyclic forms. The major transcripts in both stages carry a very short 5'-untranslated region of only 2 nucleotides length (data not shown).

**Figure 3:**
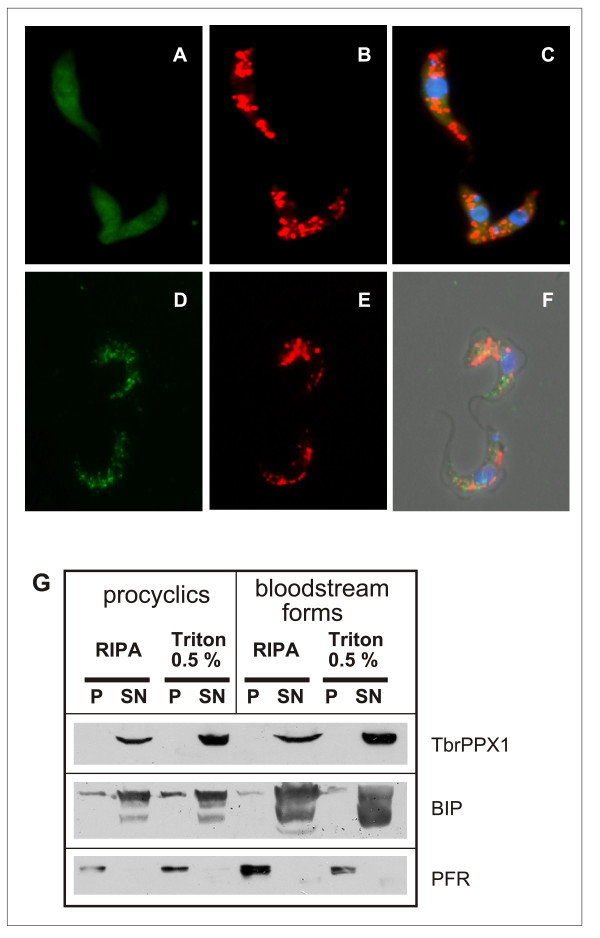
**Subcellular localization of TbrPPX1**. Panels A-C: procyclic forms. Panels D-F: bloodstream forms. Panels A and D: c-Myc-tagged TbrPPX1; panels B and E: acidocalcisomes visualized by the VH^+^-PPase-antibody; panels C and F: overlay, including DAPI staining. Panel G: Detergent fractionation of bloodstream forms and procyclic cells. Pellets (P) and supernatant fractions (SN) of cells solubilized either with RIPA buffer or with 0.5% Triton X-100. Western blots were developed with monoclonal anti-c-Myc antibody (= TbrPPX1), a polyclonal antiserum against BIP, and a polyclonal antiserum against a major paraflagellar rod (PFR) protein.

To establish the subcellular localization of TbrPPX1 in procyclic and bloodstream form trypanosomes, one allele was C-terminally tagged with a triple c-Myc tag [18]. The correct integration of the tagging construct was verified by Southern blot analysis of genomic DNA, and the expression of the tagged protein was confirmed by Western blotting. Immunofluorescence microscopy of procyclic cells showed an intense but diffuse cytosolic staining throughout the entire cell body, but not in the flagellum (Figure 3 panel A).

In the bloodstream form, staining was also found throughout the cell body, but was significantly more granular (Figure 3 panel D). Staining of the cells with an antibody against the TbV-H^+^-PPase, an acidocalcisome marker [12] visualized the well defined acidocalcisomes throughout the cell (Figure 3 panels B and E). In procyclics, the distinct localization of the acidocalcisomes clearly contrasted with the homogeneous, diffuse distribution of TbrPPX1. In the bloodstream form, both TbrPPX1 and acidocalcisomes show defined, punctate localizations, which however do not colocalize. These observations are similar to what was found with the *L. major *homologue LmPPX [14], suggesting that the protein is similarly localized in both species. No fluorescence was observed in control wild type procyclic 427 and bloodstream 221 cells incubated with mouse monoclonal antic- Myc antibody, and in control parasites incubated only in the presence of the secondary fluorescein-labelled goat anti-mouse and anti-rabbit IgG. Triton-fractionation of procyclic and bloodstream trypanosomes showed that TbrPPX1 is fully Triton-soluble and is not an integral part of the cytoskeleton (Figure 3G).

### Knocking out TbrPPX1 in procyclic trypanosomes

**Figure 4:**
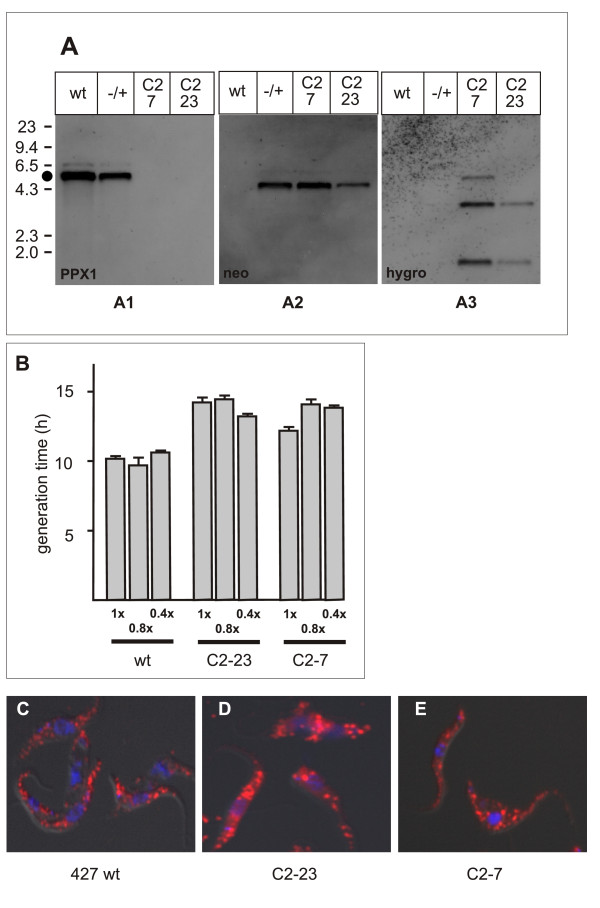
**Knocking out TbrPPX1 in procyclic forms does not affect cell growth or acidocalcisome distribution**. Panel A: Southern blot of knock-out constructs. A1: genomic Southern blot hybridized with a probe for the TbrPPX1 coding region; A2: the same blot hybridized with a probe for neomycin phosphotransferase; A3: same blot hybridized with a probe for hygromycin phosphotransferase. wt: parental strain; -/+: heterozygous knock-out; C2-7 and C2-23: homozygous knock-out clones. A lambda/HindIII size marker is indicated on the left. Black dot: position of the 5414 bp fragment containing the coding sequence for TbrPPX1. Panel B: generation time of wild type cells and the C2-7 and C2-23 clones after recovery from a 30 min incubation in normosmotic (1×) or hypoosmotic (0.8×, 0.4×) PBS buffer. Panel C-E: acidocalcisomal staining of wild type cells (panel C), and TbrPPX1 knock-out clones C2-23 (panel D) and C2-7 (panel E).

In order to assess the function of PPX1 in procyclic *T. brucei*, a gene knockout was performed. The first TbrPPX1 allele was replaced by a neomycin resistance and the second allele was replaced by a hygromycin resistance gene. The homozygous deletion of TbrPPX1 in two independent clones was confirmed by genomic PCR and by Southern blot (Figure 4A). The knock-out strains exhibited only a subtle growth phenotype. The mean generation time of the knock-out clones was determined in two independent experiments for each clone. When compared to wild type procyclic 427 cells, it was increased by 2.4 h and by 3.8 h for clones C2-7 and C2-23, respectively. Growth of wild-type cells and knock-out clones in hypoosmotic medium (0.8× and 0.4× of standard osmolarity) did not appreciably change the generation time of either strain (Figure 4B). To determine if PPX1 might be involved in regulating the cellular energy level, total cellular ATP was determined. Interestingly, the two independent knock-out clones exhibited different ATP contents, but in either case this was lower than that of wild type cells (3.84 ± 1.6 mM (n = 3) for wild type vs 3.19 ± 1.4 (n = 4) and 2.33 ± 1.0 mM (n = 3) for clones C2-7 and C2-23, respectively). DAPI staining revealed that clones C2-7 and C2-23 had a normal nucleus/kinetoplast ratio when (data not shown). The number and size of acidocalcisomes as well as their subcellular distribution seemed to remain unchanged between wild type cells and the two knock-out clones (Figure 4C-E). Similarly, the cellular polyphosphate content remained unaltered between wild-type and TbrPPX1 knock-out clones (Table 2).

**Table 2:** Polyphosphate content of trypanosomes.

	blooodstream form 221	Procyclic form 427	TbrPPX1 knock-out strain C2-23
ng polyphosphate/10^6 ^cells	2898 ± 903 (n = 3)	5712 ± 422 (n = 6)	4568 ± 1346 (n = 8)

relative standard error	18.0%	12.6%	10.4%

### Bloodstream trypanosomes are not sensitive to RNAi against TbrPPX1

**Figure 5:**
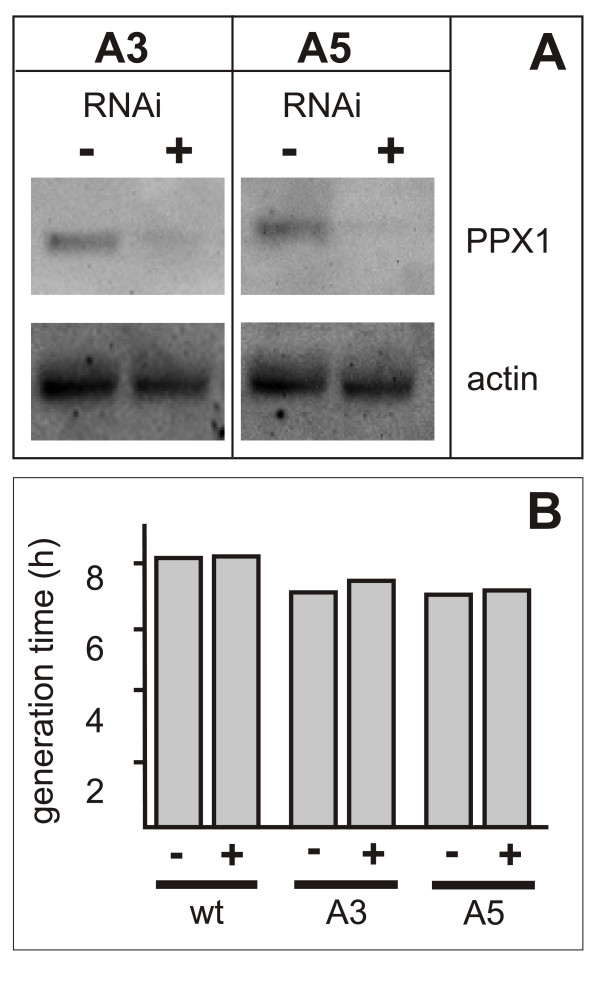
**RNAi against TbrPPX1 does not affect proliferation of bloodstream forms in culture**. Panel A: Northern blot of two independent bloodstream form clones at 48 h after induction of RNAi. Panel B: determination of generation times in the presence and absence of tetracycline. wt: NYSM parental strain, A3, A5: two independent clones expressing RNAi against TbrPPX1. The figure represents one of two independent experiments.

Attempts to construct viable TbrPPX1 knock-outs in bloodstream forms failed repetitively. Therefore, RNAi was attempted as an alternative procedure. Northern blot analysis of TbrPPX1 RNAi strains in the presence or absence of 1 μg/ml tetracycline demonstrated that the RNAi constructs were functional and that the level of target mRNA was strongly reduced (Figure 5A). Nevertheless, RNAi-mediated gene knock-down of TbrPPX1 in the presence of tetracycline did not result in a significant change of growth rates in culture (Figure 5B). No changes in cell morphology could be observed. When RNAi was induced for 48 h against PPX1 in both clones, A3 and A5, no change in either ATP concentration or polyphosphate content was observed. Both clones were then used in two independent experiments to infect mice that had received tetracycline in their drinking water to induce RNAi. No difference in virulence was observed between mice receiving tetracycline and control animals. In conjunction, these data suggest that TbrPPX1 may not be an essential gene in bloodstream form *T. brucei*, neither in cell culture nor during an in vivo infection.

### Characterization of recombinant TbrPPX1

TbrPPX1 was expressed in *E. coli *BL21(DE3) cells as a fusion protein with either an N-terminal GST tag or an N-terminal MBP tag, using the pGST- or pMBP parallel3 vectors [19]. Induction of protein expression with 0.4 mM IPTG overnight at 15°C resulted in mostly soluble fusion protein. The recombinant proteins were isolated by passage over glutathione- or amylose-resin. Both recombinant proteins migrated with the expected molecular masses (TbrPPX1: 42.8 kDa; GST: 26.2 kDa; MBP: 41.2 kDa). Initial activity measurement using pentasodium triphosphate as a substrate demonstrated that the GST-fusion protein was active, while the MBP fusion construct was completely inactive. In contrast to what was observed with LmjPPX1 [14], recombinant TbrPPX1 was stable after purification, and could be frozen and thawed repeatedly without loss of activity when kept in elution buffer containing 10% glycerol and 0.5 mM MgCl_2_. As expected from its sequence, TbrPPX1 proved to be an exopolyphosphatase. Its K_m _for pentasodium triphosphate as a substrate is 27.2 ± 4.2 μM (n = 3), and its k_cat _is 8.1 ± 1.5 s^-1 ^(n = 3).

**Figure 6:**
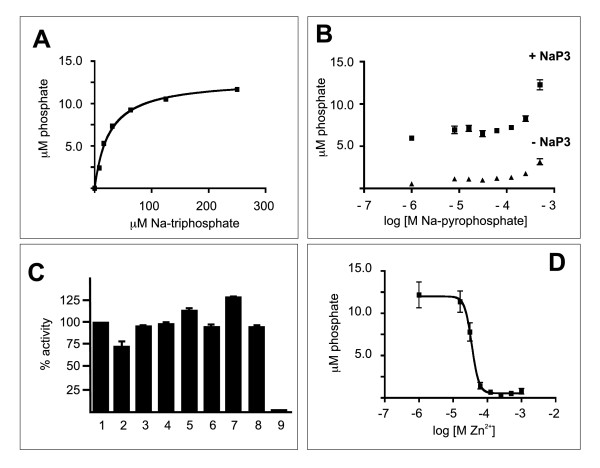
**Characterization of recombinant TbrPPX1**. Panel A: Michaelis-Menten kinetics with pentasodium triphosphate as substrate. Each assay point was done in triplicate (standard deviations are too small to be visible in the graph). A representative graph of three independent experiments is shown. Panel B: Sodium pyrophosphate is neither a substrate for, nor an inhibitor of TbrPPX1. Enzyme reactions were run in the absence (triangles) or presence (squares) of 100 mM petasodium triphosphate, and with increasing concentrations of sodium pyrophosphate. Panel C: Influence on TbrPPX1 activity (at 100 μM sodium pentaphosphate) by 1: H_2_O (control); 2: 1 mM sodium pyrophosphate; 3: 1 mM cAMP; 4: 1 mM of each dATP, dCTP, dGTP and TTP; 5: 300 mg/ml tRNA; 6: 100 u/ml heparin; 7: 200 u/ml heparin, 8: 10 mM arginine, and 9: 10 mM EDTA. Panel C: Inhibition of TbrPPX1 by Zn^2+ ^in the presence of 1 mM MgCl_2_.

Sodium pyrophosphate (Figure 6B) and polyphosphate (average length ~ 17) are neither substrates nor inhibitors. The activity of TbrPPX1 is entirely dependent on divalent cations, and it is not affected by cAMP, deoxynucleoside triphosphates, ATP, sodium pyrophosphate, by basic amino acids that are enriched in the acidocalcisomes such as arginine, or by long polyanions such as heparin or RNA (Figure 6C). Also, TbrPPX1 is not inhibited by a series of cyclic nucleotide phosphodiesterase inhibitors such as Ro-20-1724, sildenafil, zaprinast, papaverine or etazolate, or the sodium salts of vanadate, fluoride or sulfate. Zn^2+ ^is a strong inhibitor with an IC_50 _value of 21.3 ± 18.2 μM (n = 3) when the reaction is run in the presence of 1 mM MgCl_2 _(Figure 6D).

### Lack of cAMP-PDE activity in endogenous TbrPPX1

**Figure 7:**
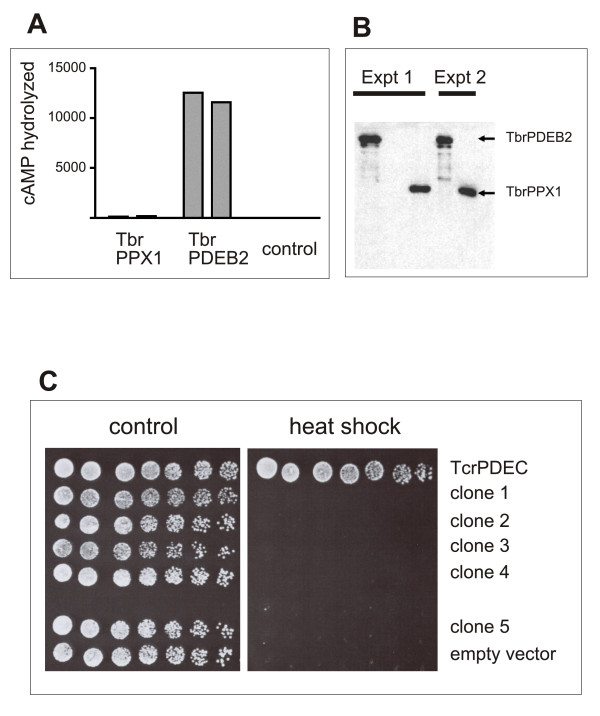
**TbrPPX1 does not exhibit cyclic nucleotide phosphodiesterase activity**. Panel A: PDE activity of immunoprecipitates from procyclic cells expressing c-Myc-tagged TbrPPX1 and TbrPDEB2, respectively, and from wild type procyclics. The results of two independent experiments are given for each. Panel B: Western blotting demonstrating that the respective proteins are expressed and present in the lysates used for immunoprecipitation. Panel C: Complementation of PDE-deficient *S. cerevisiae*. First row: strain expressing *T. cruzi *PDEC (positive control); rows 2 - 6: clones expressing TbrPPX1; row 7: strain carrying the empty vector (negative control). Each row from left to right: serial 10-fold dilutions, 5 μl spotted.

Human prune, a closely related exopolyphosphatase [9] was reported to also contain a cAMP-specific phosphodiesterase activity [17]. If true, this finding would have the potential to profoundly alter the current paradigms of eukaryotic cAMP signaling, which are largely based on class 1 cyclic nucleotide-specific phosphodiesterases as the only mechanisms for rapidly disposing of cAMP [20]. To investigate if TbRPPX1 might show a similar activity, recombinant TbrPPX1 was tested for possible cAMP phosphodiesterase activity. No cAMP hydrolysis could be detected. To ascertain that the observed lack of PDE activity was not due to the fact that a recombinant protein was used, TbrPPX1 was also analyzed after immunoprecipitation from trypanosome lysates. 3× c-Myc tagged TbrPPX1 protein from ~ 1.5 × 10^7 ^procyclic cells was immunoprecipitated, and the precipitates were assayed for PDE catalytic activity. Control precipitates were done with lysates from cells expressing the 3× c-Myc tagged phosphodiesterase TbrPDEB2. The results demonstrate that immunoprecipitated TbrPPX1 does not exhibit detectable PDE-activity while such an activity is easily detected with an immunoprecipitated control PDE (Figure 7A). These findings agree with those obtained with the recombinant protein, and they support more recent experiments with human prune that also failed to detect an intrinsic PDE activity [9].

A third approach attempting to demonstrate phosphodiesterase activity in TbrPPX1 used a very sensitive in-vivo complementation system for phosphodiesterase activity [21]. The assay consists in the reversion of a phosphodiesterase-deficient, and therefore heatsensitive strain of *S. cerevisiae *to heat-shock resistance through the introduction of a heterologous phosphodiesterase activity. The assay is exquisitely sensitive for cAMP-phosphodiesterase activity and allows its detection even under conditions where no activity can be biochemically measured in the corresponding yeast cell lysates [21,22]. Western blot analysis of the yeast lysates demonstrated that TbrPPX1 is stably expressed in all of the five yeast clones tested (data not shown). Nevertheless, TbrPPX1 did not restore the heat shock resistance phenotype to the PDE-deficient indicator strain (Figure 7B), whereas TcrPDEC, a control phosphodiesterase from *Trypanosoma cruzi *[23], did fully restore this phenotype. The results of these complementation experiments further support the view that TbrPPX1 protein does not contain cAMP-phosphodiesterase activity.

## Discussion

The currently available genomes of kinetoplastids all harbor genes for three different groups of polyphosphatases that belong to subfamily 2 of the DHH superfamily. Group 1 (of which TbrPPX1 is a member) comprises the cytosolic exopolyphosphatases (EC 3.6.1.11) that are related to those e.g. of the ascomycota such as *S. cerevisiae*. Group 1 enzymes have been characterized in *T. cruzi *[15] and in *L. major *[14], and preliminary report has indicated a corresponding activity in *T. brucei *[16]. Group 2 contains predicted acidocalcisomal pyrophosphatases (EC 3.6.1.1) that are specific for the kinetoplastids, and group 3 consists of putative inorganic pyrophosphatases (EC 3.6.1.1) for which no experimental evidence is yet available. The two latter groups share extensive sequence identity among themselves as well as with the fungal inorganic pyrophosphatases throughout their catalytic domains. The group 2 enzymes (the acidocalcisomal pyrophosphatases) all contain an additional N-terminal extension of 180 - 200 amino acids. These extensions are highly similar between all kinetoplastids species and may contain the information for their acidocalcisomal localization. In *T. brucei*, the group 2 pyrophosphatase TbrVSP1 has been characterized experimentally [12,13].

The cytosolic exopolyphosphatases (group 1) enzymes are encoded by single-copy genes in all kinetoplastid genomes, with the exception of *T. cruzi *whose genome contains three such genes. TbrPPX1 of *T. brucei *encodes a protein of 383 amino acids, with a calculated molecular mass of 42.8 kDa and a pI of 5.39. Interestingly, no gene for endopolyphosphatases have yet been detected in the kinetoplastid genomes. These might not be required since the average length of the polyphosphates in these organisms is so short (only 3-4 residues per chain in *T. cruzi *[3]) that they could be efficiently handled by exopolyphosphatases alone. In addition, the demonstrated capacity of pyrophosphatase TbrVSP1 to slowly hydrolyze even long-chain polyphosphates might be sufficient for taking care of the occasional long-chain polyphosphate.

Analysis of the subcellular localization of TbrPPX1 demonstrated that it is present throughout the cell body, in agreement with the PPX1 enzymes being cytosolic exopolyphosphatases in other organisms as well. In procyclic trypanosomes, it is homogeneously distributed throughout the entire cytoplasm, with no evidence for specific co-localization with the acidocalcisomes. This is similar to the subcellular localization observed with its homologue in *L. major *[14]. In the bloodstream form, TbrPPX1 is localized in more granular structures throughout the cytoplasm, suggesting that its subcellular organization might be lifecycle stage dependent. Nevertheless, these granules exhibit no specific co-localization with the acidocalcisomes. In both stages, TbrPPX1 is excluded from the flagellum. Upon cell fractionation of either procyclic or bloodstream cells with the non-ionic detergent Triton X-100, TbrPPX1 partitions quantitatively into the soluble phase, demonstrating that it is not firmly associated to cytoskeletal structures in either life cycle stage. This is in agreement with the observation that TbrPPX1, similar to LmPPX [14], lacks an N-terminal signal sequence, suggesting that it does not enter the endoplasmic reticulum-mediated secretory pathway, but is synthesized on free polysomes and then kept in the cytosol.

TbrPPX1 is an active exopolyphosphatase that accepts inorganic pentasodium triphosphate as a substrate, but neither nucleoside triphosphates nor inorganic pyrophosphate. The marked inhibition of TbrPPX1 by Zn^2+ ^ions even in the presence of a large excess of Mg^2+ ^is reminiscent to what was reported for its *L. major *[14] and *T. cruzi *[15] homologues.

Several experimental approaches have demonstrated that TbrPPX1 definitely does not contain an endogenous cAMP-phosphodiesterase activity. This is in agreement with recent similar findings with human prune [9] for which such an activity had initially been postulated [17]. Also, the exopolyphosphatase activity of TbrPPX1 is not inhibited by several inhibitors with specificities against different human cyclic nucleotide-specific phosphodiesterases. These findings support the central paradigm of cAMP signaling in eukaryotes which posits that the cyclic nucleotide-specific phosphodiesterases represent the only mechanism for a rapid disposal of cAMP.

TbrPPX1 is not essential in *T. brucei*, neither in the procyclic nor in the bloodstream form. Gene ablation by genetic knock-out or knock-down by RNAi only slightly prolonged the generation time. Furthermore, in-vivo RNAi in a mouse model did not abolish the virulence of two independent RNAi clones. The absence of a dramatic phenotype is in agreement with the observation that the overall polyphosphate content of wild type versus TbrPPX1-knockout cells was not changed, suggesting that TbrPPX1 is not involved in the quantitative management of polyphosphate stores. The overall polyphosphate content measured for *T. brucei *in this study is in good agreement with earlier findings with *T. cruzi *[11]. The observed lack of a significant phenotype in the TbrPPX1 knock-outs is also in good agreement with similar findings in *S. cerevisiae*, where inactivation of the exopolyphosphatases PPX1 and PPN1 did not prevent the utilization of polyphosphates as a phosphate reserve [24]. Knocking out PPX1 does only slightly downregulate the cellular ATP content, indicating that PPX1 is not a major contributor to the cellular energy balance. These findings are in marked contrast to what was observed with a group 2 enzyme, the acidocalcisomal pyrophosphatase TbrVSP1 [12], which clearly is an essential enzyme, also for in vivo infections. In conclusion, the cytosolic exonuclease TbrPPX1 seems to play only a modulatory role in the overall polyphosphate metabolism of *T. brucei*, and it plays no significant role in the overall energy balance of trypanosomes. It might possibly fulfil a specific role in handling local cytoplasmic pools of polyphosphates that are quantitatively minor compared to the acidocalcisomal polyphosphate stores.

Alternatively, TbrPPX1 might be crucial to optimize the phosphate metabolism in specific situations or life cycle stages, but might have no major role for cell proliferation during the easy life in the affluent environment of a culture medium or a mammalian host.

## Conclusions

The genomes of all kinetoplastida sequenced to data contain a similar set of genes that code for polyphosphatases. The group 1 enzymes, including TbrPPX1, are exopolyphosphatases [[14-16], this study]. Groups 2 and 3 represent pyrophosphatases, where the group 2 enzymes are located in the acidocalcisomes [12,13,35], while the group 3 enzymes are most likely cytoplasmic, though no experimental data on any of them are available yet. TbrPPX1 is an exopolyphosphatase which is specific for inorganic polyphosphate, and it exhibits a K_m _value of around 30 μM for pentasodium triphosphate. It does not hydrolyze, nor is it inhibited by organic polyposphates such as ATP, or by Na-pyrophosphate. The enzyme activity is completely inhibited by EDTA, and it is also strongly inhibited by Zn^2+^, even in the presence of a large molar excess of Mg^2+^. An important aspect in the context of intracellular signaling is the observation that TbrPPX1 does not exert cyclic nucleotide phosphodiesterase activity, as has been postulated earlier for the human prune exopolyphosphatase [17]. While the current study was in progress, this claim for the human enzyme has been essentially retracted [9]. Immunofluorescence staining demonstrated that TbrPPX1 is localized throughout the cytoplasm, without a recognizable association with subcellular structures. A genetic knockout of TbrPPX1, or its knock-down via RNA interference do not produce dramatic phenotypes. In agreement, the overall polyphosphate content in the various mutants is not significantly different from the respective wild type cells. Combined with the observation that TbrPPX1 is localized in the cytoplasm and is not associated with the major polyphosphate stores, the acidocalcisomes, the data indicate that TbrPPX1 may not be involved in the phosphate/polyphosphate metabolism on a quantitative scale. Rather, it might exert modulatory functions based on cytoplasmic polyphosphate that cannot be identified by simple genetic knock-out experiments.

## Methods

### Trypanosome cell culture

Procyclic *T. brucei *427 cells were cultured at 27°C in complete SDM79 medium [25] supplemented with 5% (v/v) heat-inactivated foetal calf serum (FCS). Bloodstream forms of the monomorphic strain 221 (Mitat 1.2) were cultured in HMI-9 medium [26] supplemented with 10% (v/v) FCS at 37°C in a 5% CO_2 _atmosphere.

### Sequence searches and alignments

The TbrPPX1 gene from *T. brucei *was identified by TBLASTP search with the human prune amino acid sequence [GenBank: NP_067045] as a query. Blast-searching GeneDB http://www.genedb.org revealed a single predicted protein [GeneDB:Tb09.160.1950]. This sequence was then used for iterative searches of other kinetoplastid genomes for related proteins (*T. brucei, T. cruzi, T. vivax, T. congolense, L. major, L. infantum, L. braziliensis*, and *L. tarentolae*). Multiple alignments of amino acid sequences were obtained using ClustalW v1.82, Jalview and BioEdit v7.0.5 software using the similarity matrix BLOSUM62.

### Cloning and sequencing

The open reading frame of TbrPPX1 gene (1152 bp) was PCR amplified from genomic DNA of procyclic *T. brucei *427 using the primers TbLw43f (5'-CATATGAGGATCCAAATGACGGCAGTGGTGAATGAGTTC-3') and TbLw43r (5'- CTCGAGGCGGCCGCTTACAAATTGTTCCACACTGACAAAAAACTAG-3').

Restriction sites for NdeI and BamHI and for XhoI and NotI used for subsequent cloning are underlined. The resulting PCR product was cloned into the pCR2.1-TOPO vector (Invitrogen) and sequenced. Comparison of the amplified TbrPPX1 DNA sequence from *T. brucei *427 gDNA with the DNA sequence of the corresponding locus Tb09.160.1950 in the *T. brucei *427 genome sequence database revealed a few sequence differences at the DNA level, but none in the predicted amino acid sequence.

### Southern and Northern Blot Analyses

Genomic DNA from procyclic and bloodstream *T. brucei *cell lines was digested with the appropriate restriction enzymes, separated on a 1% agarose gel and transferred to a positively charged nylon membrane (Roche). Digoxigenin-labelled DNA probes were generated using the PCR DIG probe synthesis kit (Roche). Hybridization probes were amplified with the following primers: For the TbrPPX1 open reading frame: primers TbLw43f and TbLw43r (see above); for the G418 resistance gene: the single primer Fwneo/Rewneo (5'-CTGCCCATTCGACCACCAAGC-3') and for the hygromycin resistance gene the primer pair Fwhygro (5'-GATGTAGGAGGGCGTGGATA-3') and Rewhygro (5'-TTGTTCGGTCGGCATCTACT-3'). In order to achieve a minimal hybridization background, the DNA templates for the PCR reactions were excised from the respective plasmid vectors and further purified by gel extraction (QIAquick Gel Extraction Kit, Qiagen).

Blots were prehybridized for 6 h in DIG Easy Hyb buffer (Roche) and were hybridized at 42°C overnight in the same buffer containing 20 ng/ml of DIG-labelled probe. High stringency washes were done in 0.5 × SSC, 0.1% SDS at 68°C twice for 15 min. Hybridization signals were detected with an alkaline phosphatase-conjugated anti-DIG antibody (Roche) and the CDP-Star substrate (Roche) and visualized on a LAS-1000 Image Reader (Fuji).

For Northern blot analysis, total RNA from procyclic and bloodstream cells was denatured in 50% (v/v) DMSO, 4% (v/v) deionised glyoxal and 10 mM sodium phosphate, pH 6.85, for 5 min at 50°C and separated on a 1% agarose gel in 10 mM sodium phosphate. RNA was transferred to positively charged nylon membranes (Roche) by capillary force. Prehybridization and hybridization with the DIG-labelled probes were done as described above, but at a hybridisation temperature of 50°C. High stringency washes and hybridisation signal detection were done as described above. A hybridization probe specific for α-actin was generated with primers Actinf (5'-CCGAGTCACACAACGT-3') and Actinr (5'-CCACCTGCATAACATTG-3') for the normalization of all blots. Signals were recorded by a luminescent image analyzer (image reader LAS1000; Fuji) and analyzed and quantified with image analyzer software Aida v. 3.11.

### Generation of transgenic trypanosome cell lines

For deletion of the TbrPPX1 locus in procyclic cells, the 5' UTR and the 3' UTR sequences of TbrPPX1 gene were amplified by PCR from genomic DNA with High Fidelity Polymerase (Roche), using the primer pairs Tbprune_5UTRf (5'- GGTACCTGGCAGTTGTTAGTGAATAAGAAC-3' (KpnI)) andTbprune_5UTRr (5'- AAGCTTTATCTTAAGGCCGGAAAGTG-3' (HindIII)) for the 5'-UTR, andTbprune_3UTRf (5'-GGATCCGACCATTTTGTTATGTTGATCTGTC-3' (BamHI)) and Tbprune_3UTRr (5'-GAGCTCGCACTCAACCAGACTCGTTACTAG-3' (SacI)) for the 3'-UTR. The fragments were sequentially cloned into the KpnI/HindIII and BamHI/SacI sites flanking a neomycin or hygromycin resistance cassette in the pBluescript II KS+ phagemid, resulting in the pBS-neo and pBS-hygro TbrPPX1 KO-plasmids. The constructs were released from the plasmid DNAs by digestion with KpnI/SacI, ethanol precipitated, and transfected into procyclic 427 cells. Both TbrPPX1 alleles were replaced by successive transformations using the two antibiotic resistance cassettes. Selection of transformants was done with 15 μg/ml neomycin and 25 μg/ml hygromycin. The correct integration of neo and hygro-dKO was monitored by Southern blotting.

### Construction of an RNAi cell line

To generate the TbrPPX1 RNAi construct, a fragment of the TbrPPX1 gene (bp 645-914 of the open reading frame) was PCR amplified from genomic DNA with the Expand High Fidelity^® ^PCR system (Roche) using the following two primers (HindIII, BamHI and XbaI, XhoI sites underlined): Prune_pMP10-f (5'-CAGCAAGCTTGGATCCGACTACCTGACGGGCATGTT-3') and Prune_pMP10-r (5'-CCTCTAGACTCGAGACCAGCGAAGGTCAAGAGAA-3'). The fragment was cloned into the pMP10 RNAi vector plasmid (a derivate pLEW100) containing the appropriate restrictions sites. For stable transfection via integration into an rDNA spacer region, the RNAi construct was linearized by NotI digestion, ethanol precipitated and transfected into the bloodstream form NYSM single marker cell line [27]. Selection was done with 1 μg/ml neomycin and 0.1 μg/ml phleomycin. RNAi was induced with 1 μg/ml tetracycline.

### In situ tagging of TbrPPX1 in procyclic and bloodstream forms

To generate c-Myc-tagged TbrPPX1 in procyclic and bloodstream form trypanosomes, the entire open reading frame of TbrPPX1 (without the stop codon; bp 1-1152), as well as bp 9-1001 of the 3'UTR were amplified, using the following primer pairs (restriction sites underlined): Prune-ORFtag-f (5'-ATGGTACCATGACGGCAGTGGTGAATGAGTT-3', KpnI), Prune-ORFtag-r (5'-TACTCGAGCAAATTGTTCCACACTGACAAAAAAC-3', XhoI), Prune-3UTRtag-f (5'-ATGGATCCGACCATTTTGTTATGTTGATCTGTC-3', BamH1) and Prune-3UTRtag-r (5'-ATTCTAGATCTCGGTTAGAGCCTCTAACTCT-3', XbaI). The PCR products were ligated into vector pMOTag33 M [18]. The final construct was digested with KpnI and NotI, ethanol precipitated and transformed into procyclic form 427 and bloodstream form of strain 221 *T. brucei *cells. Transfectants were selected with 15 μg/ml (procyclics) and 1 μg/ml (bloodstream form) neomycin and were verified by Southern blotting and expression analysis.

### Western Blot analysis

Samples were diluted 1:1 in a 1.25 × SDS sample buffer (4% SDS, 20% Glycerol, 10% 2-mercaptoethanol, 0.004% bromphenol blue, 0.125 M Tris HCl), boiled for 5 min, and then applied to a 12% SDS-PAGE gel. Proteins were transferred onto Immobilon-P membranes and immunostained with mouse monoclonal anti-c-Myc 9E10 antibody (Santa Cruz; dilution 1:1000). Immunoreactivity was detected by chemiluminescence using horseradish peroxidase conjugated rabbit anti-mouse IgGs and an ECL™ Western Blotting System substrate (Amersham Biosciences).

### Triton-X-100 fractionation

5 × 10^7 ^trypanosomes were washed once in PBS and lysed on ice for 10 minutes in PBS + 0.5% Triton-X 100 or with RIPA buffer (50 mM TrisHCl, pH 8.8, 150 mM NaCl, 1% NP-40, 0.5% deoxycholate, 0.1% SDS) supplemented with protease inhibitor (Roche complete mini^®, ^EDTA-free). The cell lysate was centrifuged 15 minutes at 15'700 g (4°C), and supernatant and pellet fractions were analyzed by Western blotting.

### Immunofluorescence microscopy

For immunofluorescence microscopy, trypanosomes were centrifuged from culture medium at 2,000 × g. Tagged 427 procyclic wild type cells were washed in PBS and fixed with 4% formaldehyde in PBS (w/v) for 15 min at room temperature. The fixed cells were allowed to adhere to polylysine-coated well slides (Erie Scientific Company) for 20 min, and were then permeabilized with prechilled (-20°C) methanol for 10 min. Slides were washed for 5 min in PBS + 0.1 M glycine and for another 5 min in PBS. Blocking was done with PBS + 2.5% BSA (w/v) for 1 h. Tagged bloodstream parasites were resuspended in cold PBS and were allowed to adhere to polylysine-coated well slides (Erie Scientific Company) for 10 min and fixed with 4% formaldehyde in PBS (w/v) for 10 min at room temperature. After washing the cells 3 × 5 min with 500 ul cold PBS, the cells were permeabilized with 0.5% Triton X-100 in PBS for 2 min. Slides were washed 3 × 5 min with cold PBS and then blocked with PBS containing 2% BSA (w/v) for 60 min. The following primary antibodies were used for both cell lines: mouse anti-c-Myc 9E10 (Santa Cruz), dilution 1:300; rabbit anti-TbV-H^+^PPase (visualization of acidocalcisomes, a gift of Théo Baltz, University of Bordeaux II, France; dilution 1:500); Secondary antibodies were Alexa Fluor 488 or 594 conjugated goat anti-mouse or goat anti-rabbit (Molecular Probes; highly cross-absorbed, dilution 1:750). DAPI-staining was done with Vectashield mounting medium with DAPI (Vector Laboratories). Coverslips were mounted with Vectashield mounting medium containing DAPI (Vector Laboratories) and images were obtained using a LEICA DM 6000B microscope.

### Hypoosmotic treatment

Wild-type cells and knock-out clones were subjected to hypoosmotic treatment using a published procedure [28]. Briefly, exponentially growing cultures were centrifuged for 5 min at 3000 rpm. Individual cell pellets were suspended in PBS diluted with H_2_O to 1×, 0.8× and 0.4× regular strength, and were incubated at 27°C for 30 min. Cells were then collected by centrifugation for 10 min at 2,500 rpm, resuspended in regular SDM-79 medium and their density was adjusted to 2 × 10^6 ^cells/ml. Cell density was again determined and slides for immunofluorescence were prepared after 24 h incubation.

### ATP determination

For the determination of intracellular ATP, triplicate aliquots of 5 × 10^6 ^cells were centrifuged for 5 min at 6000 rpm. The cell pellet was suspended with 150 μl cold 1.4% perchloric acid. After incubation for 30 min on ice, 30 μl of 1N KOH were added. After incubation on ice for an additional hour, samples were centrifuged for 20 min at 13,500 rpm. 150 μl of the resulting supernatant were withdrawn for further analysis. 10 μl aliquots of such supernatant were then analyzed using the ATP Bioluminescence Assay Kit CLS II (Roche) according to the instructions of the supplier. To calculate intracellular ATP concentrations, cell volumes of 96 ± 8 μm^3 ^(9.6 × 10^-14 ^l) for procyclics and 53 ± 3 μm^3 ^(5.3 × 10^-14 ^l) for the bloodstream form (Markus Engstler, University of Würzburg, FRG; personal communication) were assumed.

### Polyphosphate determination

Total cellular polyphosphate was determined according to published procedures [29,30]. Cells (2 - 5 × 10^6^) were centrifuged, the supernatant was carefully withdrawn and the cell pellets were snap-frozen and stored at - 70°C. Polyphosphates were extracted by incubating the cell pellets with 1 ml HE buffer (25 mM HEPES, pH 7.6, 1 mM EDTA) for 30 min at 85°C, with intermittent vortexing. After cooling, the extracts were centrifuged for 10 min at 8000 rpm, and the supernatant was collected for polyphosphate determination. Fluorescence assays were performed in white microtiter plates. Five to 50 μl of supernatant were adjusted to 200 μl/well with HE buffer. After adding 20 μl of 100 μM DAPI (2-(4-Amidinophenyl)- 6-indolecarbamidine dihydrochloride; Sigma D9542; dissolved in H_2_O), the plates were vibrated for 20 min at room temperature. Fluorescence was then measured at 415 nm_EX _and 540 nm_EM_. The fluorescence signal remained stable over at least several hours. Standard curves (0 - 2000 ng/ml, in HE buffer) were constructed using polyphosphate (Aldrich, cat nr. 30,555-3) with an average chain length of 17.

### Protein expression and purification of recombinant TbrPPX1

To produce a GST-TbrPPX1 or MBP-TbrPPX1 fusion proteins, the previously constructed TOPO-TbrPPX1 plasmid was cleaved with BamHI and NotI, and the resulting fragment inserted into the pGST- or the MBP parallel3 vectors [19]. The final plasmids were verified by DNA sequencing and transformed in *Escherichia coli *BL21(DE3) cells. The cells were grown in Terrific Broth (TB) medium [31] at 37°C with constant shaking. IPTG was added to a final concentration of 0.4 mM when OD_600 _reached 0.5. Cells were further grown at 15°C and harvested 18 h after IPTG induction by centrifugation at 4000 rpm for 20 min. The pellets were resuspended in homogenisation buffer (140 mM NaCl, 20 mM HEPES, pH 7.4) containing the Roche complete^® ^protease inhibitor cocktail, and were lysed with a French Press at 20,000 psi. The cell lysate was centrifuged at 10,000 g for 30 min to remove any insoluble material. The MBP-fusion protein was purified by affinity chromatography on an amylose-resin and eluted with 10 mM maltose in 140 mM NaCl, 20 mM HEPES, pH 7.4. The GST-TbrPPX1 protein was purified using a glutathione sepharose resin (Clontech). The protein was eluted with 10 mM glutathione in 140 mM NaCl, 20 mM HEPES, pH 7.4. Fractions were analyzed on 12% SDS-PAGE gels, followed by silver or Coomassie staining. Positive fractions were pooled and frozen in aliquots at -70°C in elution buffer supplemented with 10% glycerol and 0.5 mM MgCl_2_.

### Enzymatic activity of recombinant TbrPPX1

Polyphosphatase activity was determined in 50 μl reactions containing 50 mM HEPES, pH 7.8, 50 μM EGTA, 1 mM MgCl_2 _and 20 - 40 nM enzyme. The standard substrate was inorganic pentasodium triphosphate (Sigma, cat nr 72061). Reactions were run at 30°C for 60 s and were stopped by the addition of 100 μl BioMol Green phosphate detection solution (BioMol GmbH, Germany, cat nr AK-111). Absorbance was determined at 620 nm. Every reaction was done in triplicate, plus a control reaction that did not contain enzyme. Values from this control were subtracted as background.

cAMP phosphodiesterase activity was determined essentially as described [32]. Briefly, the assay mixture (final volume 100 μl) contained 30 mM TrisHCl, pH 7.4, 5 mM MgCl_2_, 100 μM EGTA, and 0.5 μM cAMP, including 30,000 cpm ^3^H-cAMP. Incubations were performed for 15 min at 37°C and reactions were terminated by adding 25 μl of 0.5 M HCl per well. After 10 min incubation on ice, 30 μl of 1 M Tris base were added for neutralization. 10 μl (0.2 units) of alkaline phosphatase were then added. Following an incubation for 15 min at 37°C, the assay mixtures were loaded onto QAE-Sephadex A25 columns (1 ml bed volume). Columns were eluted with 1.6 ml of 30 mM ammonium formate (pH 6.0). The eluate was collected into 10 ml Ultima Flo AF scintillant (Perkin Elmer), and radioactivity was determined by scintillation counting. Results were corrected for blank values (measured in the presence of denatured protein) that were always below 2% of total radioactivity. During all assays, enzymatic degradation of cAMP did not exceed 25% of the substrate.

### In vivo RNAi

For infection experiments, female NMRI mice of ca. 12 weeks of age were used (Charles River, France). Animals were given feed and water ad libitum. Three days before infection and throughout the experiment, one group of animals received 0.5 mg/ml doxycycline (Sigma D9891) in deionized drinking water [33]. The doxycycline was replaced daily. Water uptake was monitored daily and was not different between animals receiving water only and those receiving water with doxycyline (ca. 4.5 ml per mouse per 24 h). Animals were infected by intraperitoneal injection of two independent RNAi clones, at parasite loads of 10^5 ^(experiment 1) or 10^6 ^(experiment 2) trypanosomes per animal. Starting at day 3 after infection, 2 μl tail blood was collected into 48 μl 0.85% NH_4_Cl, 10 mM TrisHCl, pH 7.5 on ice. Parasites were counted in a Neubauer chamber. All animal experimentation was done under a permit and according to the rules and regulations of the government committee on animal experimentation.

### Functional complementation of a PDE-deficient yeast mutant

The complete coding sequence of the TbrPPX1 gene was cloned into the NdeI/XhoI sites of the pLT-His vector [24], transformed into the PDE-deficient *S. cerevisiae *strain PP5 (MATa leu2-3 leu2-112 ura3-52 his3-532 his4 cam pde1::URA3 pde2::HIS3 [34], plated onto synthetic complete minus leucine (SC-Leu) medium and grown at 30°C. Single colonies were picked into liquid SC-leu medium and were grown until they reached an OD_600 _of 1.5. At this point, 150 ul aliquots of the cell suspension were incubated for 5' at 52°C in a waterbath to perform the heat shock. After briefly cooling in ice, the cells were serially diluted (1 : 10 dilution steps, using 96-well plates) with SC-leu medium. Five microliters of each dilution were finally spotted onto YPD plates, and the plates were incubated at 30°C for 2 days to monitor cell survival after the heat shock. To monitor expression of the recombinant protein, yeast cells were broken in a bead-beater. Crude debris was removed by centrifugation for 6 min at 6000 rpm in a Sorvall SS-34 rotor. The resulting supernatant was then cleared by a second centrifugation for 20 min at 13,000 rpm. Most of the recombinant enzyme was recovered in this cleared supernatant, which was subsequently used for Western blotting and phosphodiesterase activity measurements.

## Authors' contributions

EL and TS conceived the project, and EL conducted most of the work. LW contributed to recombinant protein expression and PDE assays, SK provided the expertise and conducted many of the yeast experiments, FF contributed to polyphosphatase activity measurements. TS drafted and wrote the manuscript. All authors have read and approved the final text.
